# Use of complementary and alternative medicine in patients with chronic liver diseases in Germany- a multicentric observational study

**DOI:** 10.1186/s12906-024-04607-x

**Published:** 2024-09-23

**Authors:** Fleur Sophie Gittinger, Anna Rahnfeld, Elena Lacruz, Alexander Zipprich, Frank Lammert, Cristina Ripoll

**Affiliations:** 1 Martin-Luther-University Clinic Halle (-Saale), Innere Medizin I (Gastroenterology, Hepatology, Pneumology), Halle, Germany; 2 Department for Gastroenterology, Hepatology, Diabetology and Endocrinology, St. Georg Hospital, 04129 Leipzig, Germany; 3 Martin-Luther-University Clinic Halle (-Saale), Medical Epidemiology, Biometrics and Computer Sciences, Halle, Germany; 4 Jena University Hospital, Innere Medizin IV (Gastroenterology, Hepatology, Infectious diseases, Interdisciplinary Endoscopy), Jena, Germany; 5 University Clinic of the Saarland, Innere Medizin II (Gastroenterology, Hepatology, Endocrinology, Diabetology and Nutritional Sciences), Homburg, Germany; 6 Hannover Medical School, Health Sciences, Hannover, Germany

**Keywords:** CAM, Chronic liver disease

## Abstract

**Background:**

The use of Complementary and alternative medicine (CAM) in chronic liver disease (CLD) patients in Germany is unknown. This study investigated the frequency of CAM use and associated sociodemographic, clinical and personality factors in CLD patients in Germany.

**Methods:**

This is a cross-sectional multicenter study of CLD patients attending liver outpatient clinics of university hospitals in Halle(-Saale) and Homburg between 2015 and 2017. Dedicated questionnaires recorded CAM use, sociodemographic and personality factors (evaluated with the “Big five” model, “Hospital Anxiety and Depression”-, “Multidimensional Health Locus of Control”- score). Uni- and multivariate analyses assessed factors associated to CAM use.

**Results:**

Overall 378 patients were recruited, 92 (24.3%) reported to CAM use. On univariate analysis, female CAM users were older (*p* = 0.001) and more physically active (*p* = 0.002), male CAM users more often used homeopathy (*p* = 0.000), actively promoted their health (*p* = 0.010) or had UDC in their medication (*p* = 0.004). Logistic regression analysis adjusted for personality factors showed significant association of age, physical exercise (females) and satisfaction with alternative medicine (females, males) to CAM use.

**Conclusions:**

CAM use is prevalent among CLD patients in Germany and is significantly associated to satisfaction with alternative medicine (females, males), physical exercise and older age (females). Doctors should actively inquire CLD patients about CAM use, as hepatotoxicity or interaction with medication can occur.

**Supplementary Information:**

The online version contains supplementary material available at 10.1186/s12906-024-04607-x.

## Introduction

Cirrhosis is the end-stage of chronic liver disease (CLD) due to a wide variety of different noxa. In Germany, the most common causes of cirrhosis are alcohol-associated and non- alcoholic fatty liver disease with alcohol related liver disease being the most frequent [1]. In patients with chronic liver disease, one of the main elements of therapy is the treatment of the etiological cause, which frequently involves lifestyle modifications. However, adherence to these modifications may be challenging.

Complementary and alternative medicines (CAM) have been described by the National Center for Complementary and Alternative Medicine (NCCAM) as “diverse medical and health care systems, practices and products that are not presently considered to be part of conventional medicine” [2]. The use and frequency of CAM varies in different countries [3, 4], with rates of use within the general population between 40 and 62% in Germany [5, 6] and 19–62% in the US [7–10]. CAM use in Western general populations has been shown to be associated with female gender, higher income and education levels and older age [7, 11–14]. In the setting of CLD, CAM are used for the remedy of conditions potentially related to CLD as well as to target the underlying causes of CLD [12, 15]. Its use has been linked to higher levels of education and income, female gender and poorer health status either due to hospitalization for CLD or comorbidities, or poorer rating of general health by patients in the US, Canada, and Ireland [9, 12, 15–20]. As to date, there is no information about CAM use in CLD patients in Germany. Nevertheless, there is a long tradition of CAM use in Germany [21], some of which are funded by health insurance companies.

The aim of the study was to evaluate the prevalence of use of CAM in out-patients with CLD under hepatological care in an academic setting in Germany and to identify factors are associated with its use in this at-risk patient population.

## Methods

### Data source and collection

#### Study design

This study was a multicentric, cross-sectional, observational study including patients with chronic liver disease based in Germany. Approval from the ethics board of the University Halle- Saale was obtained (Ethics approval number 2017-17). All patients signed an informed consent before participation in the study.

#### Study population

All patients with chronic liver disease who attended the outpatient liver clinics of two German university hospitals between 11/2015 and 11/2017 were proposed to participate in the study. Exclusion criteria were (a) lack of chronic liver disease as defined by increased serum activities of liver transaminases or GGT for less than 6 months, (b) patients with benign liver lesions such as focal nodular hyperplasia or hepatocellular adenoma without increase in liver transaminases or GGT, (c) patients who did not agree to participate in the study.

#### Data collection

Patients who attended the outpatient clinic several times during the study period were included only once in the study. All patients underwent an interview with a study member (AR and BD), who then helped the patient complete the questionnaire at the time of a planned outpatient visit. This interview was done in a quiet place with sufficient privacy prior to the medical consultation. The study team member who performed the interview was independent of the team responsible for the medical management of the patient. The attending physician was not aware of the answers of the patient. Information regarding present or past use of CAM was collected; the definition of “complementary medicine” being health care approaches not typically found in conventional medicine used in combination with conventional medicine or “alternative medicine” when these forms of treatment were used instead of conventional medicine [2]. No differentiation was made between traditional medicine, i.e. medical practices rooted in a country´s traditions, and alternative medicine. CAM and other health promoting measures associated with liver health but also taken for other indications (e.g. general well- being, weight loss) were sourced from NCCAM (https://www.nccih.nih.gov). The questionnaire (in German only, additional file 1) was developed with the support of a psychologist (EL).

#### Collected data

The following variables were collected:

##### Medical

Etiology and stage of liver disease (CLD, compensated cirrhosis and decompensated cirrhosis) were recorded. Furthermore, the presence and amount of clinically significant comorbidities, e.g. chronic renal failure, chronic obstructive pulmonary disease, coronary heart disease and any hospital stays within the past five years, irrespective of being liver related, were documented.

##### CAM related

The type of CAM was classified as herbal medicine taken either as tea, extract or in powdered form (milk thistle, grapefruit, artichoke, grape seed extract, prickly pear, chamomile, microalgae (spirulina, chlorella), berries (Açai-, goji-, blue-, barberries), glycyrrhizin), dietary supplements (vitamins, choline). Ongoing or past use of CAM were considered. Patients were also interviewed regarding change of symptoms or clinical state related to CAM use (symptom relief, overall increase of wellbeing) and whether any information of the family doctor concerning CAM use had taken place. The source of recommendation for CAM use was documented. Finally, further means of health improvement apart from medication, such as diet, physical exercise and homeopathy, were recorded. The term “mental” referred to meditation, prayer or autogenic training, taking no further measures to improve one´s health apart from the prescribed medication (i.e. not pertaining a healthy lifestyle) was referred to as “nothing”.

##### Socioeconomic factors

Participants’ highest educational attainment, employment and marital status, and religion were recorded. No/ basic education included participants who left school before attaining at least a high school degree. However, all participants were able to provide an informed consent to take part in the study. Alcohol intake was recorded as the most widespread substance use.

##### Psychometric tests

Three psychometric tests were used to characterize the psychological traits of the participants. The tests´ internal consistency was measured with Cronbach´s alpha with values > 0.7 implying an acceptable and values over > 0.9 an excellent reliability of the test. The extent to which participants attributed their health to their own actions or to external agents was evaluated with the “Multidimensional Health Locus of Control” (MHLC) [22, 23] score, modified to the needs of this study by reducing the original three 6 item subscales to three 3 item subscales. The MHLC scale consisted of three 3- item Likert- type scales for the dimensions “internal”, “chance” and “powerful others” designed to assess the extent to which individuals attributed the outcome of their health to either one´s own behavior, chance or external agents such as doctors, with higher scores reflecting stronger beliefs. The reported reliability is moderate, Cronbach´s alpha ranging between 0.60 and 0.75 [23]. The assessment of personality traits was based on the “Big five” factor model [24] which was translated into the German language. This model consists of a list of adjectives representing different aspects of personality: Openness to experience (α = 0.77), Conscientiousness (α = 0.57), Extroversion (α = 0.78), Agreeableness (α = 0.80), Neuroticism (α = 0.74). Cronbach´s alphas for the “Big five” score were based on the US- American MIDUS national sample [25–27]. Four- item Likert- type scales were used to describe the degree of expression for each aspect of personality. Anxiety and depression levels were evaluated with the German version of the “Hospital Anxiety and Depression Score” (HADS) made up of two 7- item Likert- type scales with higher scores reflecting an increased degree of anxiety and/ or depression [28, 29]. The Cronbach´s alpha value for the German version is reported to be > 0.80 for subscale items Anxiety and Depression [29].

##### Satisfaction with standard medical care

Satisfaction with medical care concerning participants´ liver disease and specifically interaction with the attending doctor as well as satisfaction with CAM was recorded using Likert scales (ten- point scales for interaction with the attending doctor, all others with five- point scales). The results were then grouped in three groups (not satisfied/ mixed response/ satisfied).

Only a subgroup of patients answered the questionnaires (*n* = 200) as they were introduced in the study after its initiation. Comparison of the baseline characteristics among those who did and did not answer the questionnaires showed no significant differences (Additional file 2).

### Statistical analysis

Statistical analysis was performed using SSPS version 21.0 (IBM SPSS Statistics for Windows, Version 21.0. Armonk, NY: IBM Corp.). Normality of the distribution of the variables was confirmed by means of the Kolmogorov- Smirnov test. Variables with a normal distribution were age, the “Big five” factor model, HADS, and MHLC. Missing data was not imputed. A summary of the number of missing cases for each variable is included in the additional file 3. Descriptive statistics were used to characterize the study population and CAM usage. To assess the association between the given variables and CAM use, t-tests and χ² tests were used, as appropriate. A p- value of ≤ 0.05 was defined as statistically significant. Data were analyzed separately for female/ male participants to discern any gender specific associations. All variables with a p- value < 0.1 in the univariate analysis were then included in the multivariate analysis (stepwise logistic regression analysis) to identify independent predictors of CAM use. In this analysis CAM was the dependent variable. Results are reported following the STROBE (STrengthening the Reporting of OBservational studies in Epidemiology) cross- sectional studies checklist [30].

## Results

Between November 2015 and November 2017, a total of 378 patients were recruited (Halle 300, Homburg 78). Of the 378 questionnaires, in 224 (59.3%) cases all questions in the questionnaire had been fully completed, in 154 (40.7%) cases not all questions had been answered (Additional file 3).

### Sociodemographic and clinical characteristics

A total of 182/378 (48.1%) were men, and median age of the study population was 58.4 years. Table 1 presents the sociodemographic and clinical characteristics of and regular medication taken by the study population. Patients from both centers were comparable except regarding religion, etiology and severity of liver disease. Indeed, patients from Halle had more MASLD (15.0 vs. 3.8%, *p* = 0.008) and MetALD and ALD (28.7 vs. 14.1%, *p* = 0.009), whereas viral hepatitis was more frequent in Homburg (50% vs. 14.3%, *p* = 0.000). More severe liver disease was observed in Halle as shown by a higher proportion of decompensated cirrhosis (26.0% vs. 3.8%, *p* = 0.000), although cirrhosis (compensated and decompensated) in general did not prove to be more common in Halle compared with Homburg (54.3% vs. 43.6%, *p* = 0.091). Only 76 (20.1%) of patients did not have any further medical conditions, whereas 118 (31.2%) had one, 103 (27.2%) had two, 62 (16.4%) had three and 17 (4.5%) had four or more secondary illnesses besides the liver disease.

**Table 1:** Patient characteristics (demographical and clinical features)

Variables		Female	Male
Age (years)		Mean 58.4, SD 13.0	Mean 57.9, SD 13.4
Halle, n (%)		152 (77.6)	148 (81.3)
Homburg, n (%)		44 (22.4)	34 (18.7)
Etiology			
	- MetALD and ALD, n (%)	29 (14.8)	68 (37.4)
	- MASLD, n (%)	26 (13.3)	22 (12.1)
	- Virus, n (%)	43 (21.9)	39 (21.4)
	- Cholestatic, n (%)	22 (11.2)	4 (2.2)
	- Other, n (%)	76 (38.3)	49 (26.9)
Cirrhosis	None, n (%)	110 (56.1)	71 (39.0)
	Compensated, n (%)	55 (28.2)	61 (33.5)
	Decompensated, n (%)	31 (15.8)	50 (27.5)
OV, n (%)		52 (26.5)	59 (32.4)
Variceal bleeding, n (%)		10 (5.1)	15 (8.2)
Ascites, n (%)		28 (14.3)	48 (26.4)
HE, n (%)		7 (3.6)	11 (6.0)
Jaundice, n (%)		4 (2.0)	20 (11.0)
In- patient, n (%)		147 (75.0)	132 (72.5)
Comorbidities			
	- 0, n (%)	39 (19.9)	37 (20.3)
	- 1, n (%)	64 (32.7)	54 (29.7)
	- 2, n (%)	59 (30.1)	44 (24.2)
	- 3, n (%)	28 (14.3)	34 (18.7)
	- 4 or more, n (%)	5 (2.6)	12 (6.6)

In- patient: in- patient treatment for liver related causes at any time of patient history. Abbreviations: MASLD: metabolic dysfunction associated steatotic liver disease; MetALD: MASLD and increased alcohol intake; ALD: alcohol- associated liver disease; Virus: liver disease caused by viruses; Chol.: cholestatic liver disease (primary biliary cirrhosis, primary sclerosing cholangitis); Other: autoimmune liver disease, haemochromatosis, cryptogenic liver cirrhosis etc.; CLD: chronic liver disease; LC: liver cirrhosis; OV: oesophageal varices; HE: hepatic encephalopathy

### Prevalence of CAM use

Of the 378 participants in total, 92 (24.3%) used CAM as additional therapy for their CLD and 30/92 (32.6%) patients took more than one CAM. Milk thistle 70/92 (76.1%) was taken most, followed by artichoke 32/92 (34.8%), other CAM (20.7%), L- Ornithine Aspartate (18.5%), liquorice root (1.1%), grapefruit (1.1%) and bilberries/ goji- berries (1.1%) (Additional file 4). Of note, the proportion of use of CAM was comparable between both study sites (Halle 24.3%, Homburg 24.4%) (Table 2). Other measures for promotion of health that patients undertook included physical exercise in 203/378 (53.7%) and dietary measures in 142/378 (37.7%) participants, while only 35/378 (9.3%) used homeopathy (Table 3).

**Table 2:** Frequency of CAM intake in patients with selected demographical and clinical features

Variables		Female		*p*- value	Male		*p*- value
		CAM yes	CAM no		CAM yes	CAM no	
Age (years)		*Mean 63.6*, *SD 10.1*	*Mean 56.6*,* SD 13.4*	0.001	Mean 56.5, SD 17.8	Mean 58.4, SD 11.8	0.428
Halle, n (%)		39 (78.0)	113 (77.4)	0.930	34 (81.0)	114 (81.4)	0.945
Homburg, n (%)		11 (22.0)	33 (22.6)		8 (19.0)	26 (18.6)	
Etiology	- MetALD and ALD, n (%)	4 (8.0)	25 (17.1)	0.066	12 (28.6)	56 (40.0)	0.584
	- MASLD, n (%)	9 (18.0)	17 (11.6)		4 (9.5)	18 (12.9)	
	- Virus, n (%)	16 (32.0)	27 (18.5)		11 (26.2)	28 (20.0)	
	- Cholestatic, n (%)	7 (14.0)	15 (10.3)		1 (2.4)	3 (2.1)	
	- Other, n (%)	14 (28.0)	62 (42.5)		14 (33.3)	35 (25.0)	
Cirrhosis	None, n (%)	30 (60.0)	80 (54.8)	0.671	19 (45.2)	52 (37.1)	0.632
	Compensated, n (%)	14 (28.0)	41 (28.1)		13 (31.0)	48 (34.3)	
	Decompensated, n (%)	6 (12.0)	25 (17.1)		10 (23.8)	40 (28.6)	
Symptoms ass. to LC	OV, n (%)	14 (28.0)	38 (26.0)	0.785	11 (26.2)	48 (34.3)	0.326
	Variceal bleeding, n (%)	1 (2.0)	9 (6.2)	0.248	4 (9.5)	11 (7.9)	0.730
	Ascites, n (%)	6 (12.0)	22 (15.1)	0.593	9 (21.4)	39 (27.9)	0.407
	HE, n (%)	0 (0.0)	7 (4.8)	0.115	0 (0.0)	11 (7.9)	0.061
	Jaundice, n (%)	2 (4.0)	2 (1.4)	0.256	2 (4.8)	18 (12.9)	0.141
In- patient, n (%)		37 (74.0)	110 (75.3)	0.850	31 (73.8)	101 (72.1)	0.832
Comorbidities	- 0, n (%)	10 (20.0)	29 (20.0)	0.939	7 (17.1)	30 (21.4)	0.915
	- 1, n (%)	64 (32.8)	48 (33.1)		12 (29.3)	42 (30.0)	
	- 2, n (%)	59 (30.3)	45 (31.0)		11 (26.8)	33 (23.6)	
	- 3, n (%)	28 (14.4)	20 (13.8)		9 (22.0)	25 (17.9)	
	- 4 or more, n (%)	5 (2.6)	3 (2.1)		2 (4.9)	10 (7.1)	

Abbreviations: MASLD: metabolic dysfunction associated steatotic liver disease; MetALD: MASLD and increased alcohol intake; ALD: alcohol- associated liver disease; Virus: liver disease caused by viruses; Chol.: cholestatic liver disease (primary biliary cirrhosis, primary sclerosing cholangitis); Other: autoimmune liver disease, haemochromatosis, cryptogenic liver cirrhosis etc.; symptoms ass. to LC: symptoms associated to liver cirrhosis; CLD: chronic liver disease; LC: liver cirrhosis; OV: oesophageal varices; HE: hepatic encephalopathy; In- patient: in- patient treatment for liver related causes at any time of the patient´s history

**Table 3:** Frequency of conventional medication, level of education, religion and further measures taken for improvement of health and use of CAM

		Female			Male		
		CAM yes	CAM no	p- value	CAM yes	CAM no	p-value
Conventional medication	Diuretics (torsemide, spironolactone)	17 (34.0)	49 (33.6)	0.955	13 (31.0)	41 (29.3)	0.836
	ß-Blockers (propranolol, carvedilol)	22 (44.0)	60 (41.1)	0.719	12 (28.6)	40 (28.6)	1.000
	Simvastatin	6 (12.0)	23 (15.8)	0.519	4 (9.5)	7 (5.0)	0.281
	Lactulose	10 (20.0)	17 (11.6)	0.139	4 (9.5)	18 (12.9)	0.561
	Rifaximin	0 (0.0)	1 (0.7)	0.557	0 (0.0)	5 (3.6)	0.214
	UDC	11 (22.0)	17 (11.6)	0.071	9 (21.4)	9 (6.4)	0.004
	Prednisolone	8 (16.0)	11 (7.5)	0.081	3 (7.1)	8 (5.7)	0.733
	Azathioprine	3 (6.0)	7 (4.8)	0.738	1 (2.4)	10 (7.1)	0.256
	Vitamin B complex	7 (14.0)	13 (8.9)	0.304	2 (4.8)	13 (9.3)	0.350
Level of education	No/ basic education, n (%)	1 (2.0)	3 (2.1)		0 (0.0)	2 (1.4)	0.227
	High school degree, n (%)	38 (76.0)	118 (80.8)	0.744	28 (66.7)	108 (77.1)	
	College degree, n (%)	11 (22.0)	25 (17.1)		14 (33.3)	30 (21.4)	
	Currently in relationship, n (%)	37 (74.0)	96 (65.8)	0.281	29 (69.0)	100 (71.9)	0.716
	Alcohol consumption, n (%)	9 (18.0)	36 (24.7)	0.334	17 (40.5)	41 (29.3)	0.172
Religion	No, n (%)	29 (58.0)	102 (69.9)	0.259	30 (71.2)	94 (67.1)	0.596
	Christian, n (%)	20 (40.0)	43 (29.5)		12 (28.6)	43 (30.7)	
	Other, n (%)	1 (2.0)	1 (0.7)		0 (0.0)	3 (2.1)	
Further measures for health	Diet, n (%)	20 (40.0)	72 (49.3)	0.255	14 (33.3)	39 (27.9)	0.493
	PE, n (%)	***36 (72.0)***	***69 (47.3)***	0.002	24 (57.1)	73 (52.1)	0.569
	Mental, n (%)	2 (4.0)	12 (8.2)	0.317	3 (7.1)	4 (2.9)	0.205
	Homeopathy, n (%)	8 (16.0)	13 (8.9)	0.161	***11 (26.2)***	***4 (2.9)***	0.000
	Nothing, n (%)	10 (20.0)	33 (22.6)	0.701	***6 (14.3)***	***49 (35.3)***	0.010

Variables with significant p- values on univariate analysis are printed in italics/ bold lettering. Abbreviations: UDC: ursodeoxycholic acid. PE: physical exercise; Mental: any further measures involving mind- related procedures (e.g. meditation); Nothing: no further health improvement measures taken; CAM: complementary and alternative medicine

### Information of medical personnel concerning use of CAM and satisfaction with medical treatment

A little more than half of the patients [52/92 (56.5%)] informed their physician about their CAM use. The most frequently cited reason for not reporting CAM use was that this would present no additional information and the doctor might not be familiar with the substances 15/92 (16.3%), or the doctor did not specifically ask about CAM 4/92 (4.3%). Satisfaction with conventional Western medicine was high with 188/224 (8 3.9%) of patients being very satisfied; active CAM users (female 15/27 (55.6%) and male 9/21 (42.9%)) were more satisfied with alternative medicine than CAM non-users (Table 4).

**Table 4:** Satisfaction with medical treatment

Variables	Female			Male		
	CAM yes	CAM no	*p*- value	CAM yes	CAM no	*p*- value
**Satisfaction medical treatment in general**						
- Not satisfied, n (%)	0 (0.0)	2 (2.1)	0.711	0 (0.0)	2 (2.4)	0.600
- Intermediate, n (%)	9 (33.3)	28 (29.5)		7 (33.3)	21 (25.0)	
- Satisfied, n (%)	18 (66.7)	65 (68.4)		14 (66.7)	61 (72.6)	
**Satisfaction conventional medicine**						
- Not satisfied, n (%)	0 (0.0)	4 (4.3)	0.390	1 (4.8)	2 (2.4)	0.853
- Intermediate, n (%)	3 (11.1)	16 (17.0)		2 (9.5)	8 (9.8)	
- Satisfied, n (%)	24 (88.9)	74 (78.7)		18 (85.7)	72 (87.8)	
**Satisfaction alternative medicine**						
- No (previous) experience, n (%)	***11 (40.7)***	***84 (88.4)***	***0.000***	***9 (42.9)***	***79 (89.8)***	***0.000***
- Not satisfied, n (%)	***1 (3.7)***	***1 (3.7)***		***3 (14.3)***	***0 (0)***	
- Satisfied, n (%)	***15 (55.6)***	***10 (10.5)***		***9 (42.9)***	***5 (6.0)***	

Satisfaction with alternative medicine also included answers of patients who had taken CAM in the past and who at the time of the questionnaire did not take CAM anymore. Abbreviations: CAM: complementary and alternative medicine

### Univariate analysis and factors associated to CAM use

Tables 2, 3, 4 and 5 summarize the results of the univariate analysis. In women, users of CAM were significantly older: Their mean age of CAM users was 63.6 years as compared to 56.6 years in CAM non- users (*p* = 0.001, Table 2). Among females, etiology of liver disease (*p* = 0.066) tended to be different among CAM users: Here, a greater proportion of virus- caused etiology was seen with 32.0% of patients with a virus- associated etiology using CAM as compared to 18.5% of CAM non- users. No association was observed with the severity of liver disease, the number of comorbidities present or the level of education (Table 2).

**Table 5:** Psychometric scores in study population

	Variables	Female			Male		
		CAM yes; mean (SD)	CAM no; mean (SD)	p- value	CAM yes; mean (SD)	CAM no; mean (SD)	p- value
“Big five”	Agreeableness	4.56 (0.59)	4.69 (0.45)	0.089	***4.21 (0.62)***	***4.47 (0.65)***	***0.023***
	Openness to experience	3.87 (0.63)	3.86 (0.64)	0.972	3.81 (0.76)	3.87 (0.75)	0.631
	Conscientiousness	4.33 (0.63)	4.33 (0.58)	0.057	4.00 (0.70)	4.15 (0.60)	0.193
	Neuroticism	3.22 (0.48)	3.20 (0.62)	0.838	3.14 (0.65)	3.10 (0.64)	0.720
	Extroversion	4.33 (0.67)	4.28 (0.65)	0.693	***3.77 (0.77)***	***4.04 (0.77)***	***0.047***
MHLC	Internal	2.25 (1.28)	2.39 (1.33)	0.619	2.33 (1.30)	2.48 (1.23)	0.629
	Chance	1.84 (0.95)	1.75 (0.92)	0.679	***2.03 (0.96)***	***1.50 (0.99)***	***0.030***
	Powerful others	2.30 (0.76)	1.92 (0.97)	0.064	1.87 (0.93)	1.77 (0.90)	0.642
HADS	Anxiety	6.59 (2.45)	7.22 (3.54)	0.389	5.90 (2.30)	6.04 (2.79)	0.843
	Depression	7.63 (3.67)	8.03 (3.37)	0.593	7.71 (3.15)	8.48 (3.09)	0.317

Variables with significant p- values on univariate analysis are printed in italics/ bold lettering. Abbreviations: SD: standard deviation; HADS: Hospital Anxiety and Depression score; MHLC: Multidimensional Hospital Locus of Control; CAM: complementary and alternative medicine

Women reported more frequently to use physical exercise to further promote health (72.0% in CAM users vs. 47.3% in CAM non-users, *p* = 0.003; Table 2). In men, CAM users were more likely to also use homeopathy (26.2%, *p* < 0.001) as compared to 2.9% of men who just relied on homeopathy. Men who were CAM users actively tried to promote their health more frequently than non-users (85.7% vs. 64.7%, *p* = 0.010). Male patients with ursodeoxycholic acid in their regular medication were also significantly more likely to use CAM (21.4%, *p* = 0.004) than when not (6.4%), in female patients with ursodeoxycholic acid, significance was narrowly missed (CAM users 22.0%, CAM non- users 11.6%, *p* = 0.071). Both women and men who used CAM were satisfied with alternative medicine (55.6% of female users and 42.9% of male users, *p* < 0.001 for both; Table 4).

Table 5 displays the results of the psychometric tests. A trend to lower scores with certain personality traits of the “Big five” score in males was observed such as Extroversion with CAM users having a smaller mean value for extroversion than non-users (3.77 vs. 4.04, *p* = 0.047) and Agreeableness (4.21 vs. 4.47, *p* = 0.023). Male CAM users had also higher mean values in the aspect of “Chance” in the Multidimensional Hospital Locus of Control than non-users (2.03 vs. 1.50, *p* = 0.030). No personality traits were associated to CAM usage in females. Among females, etiology of liver disease (*p* = 0.066) tended to be different among CAM users- here a greater proportion of virus- caused liver disease was seen (32% of CAM users had a viral disease compared to 18.5% of CAM non- users).

### Multivariate logistic regression analysis

All variables with a p-value < 0.1 were included in the multivariate logistic regression analysis with CAM as dependent variable. Several models were built, adjusting the variables for the “Big five” score, the HADS and MHLC (Table 6) respectively. Variables significantly associated to CAM use in more than one model were age and physical exercise in females and satisfaction with alternative medicine in both sexes.

**Table 6:** Logistic regression analysis for CAM use adjusted by “Big five” score, HADS and MHLC

	“Big five” score		HADS		MHLC	
	Adjusted OR	95% CI	Adjusted OR	95% CI	Adjusted OR	95% CI
**Female**						
Age	1.136	1.060- 1.218	1.114	1.048- 1.184	1.116	1.047- 1.188
Further measures taken for health:						
Physical exercise	-	-	14.186	1.150- 174.980	18.793	1.594- 221.513
Nothing	-	-	-	-	23.353	1.177- 463.485
Satisfaction with alternative medicine	7.789	3.093- 19.618	6.678	2.876- 15.503	7.775	3.047- 19.835
**Male**						
Satisfaction with alternative medicine	4.046	1.555- 10.524	3.867	1.544- 9.683	3.311	1.333- 8.225

Only variables with significant p- values (*p* ≤ 0.050) in the logistic regression analysis are shown. Variables included in the logistic regression analysis were age, etiology, further measures taken for improvement of health, satisfaction with conventional/ alternative medicine and medical treatment in general and were then adjusted by the “Big five”, MHLC and HADS score. Abbreviations: HADS: Hospital Anxiety and Depression Score; MHLC: Multidimensional Health Locus of Control; OR: Odds ratio; CI: confidence interval

## Discussion

This study shows that in a German population approximately 25% of patients who attend university liver clinics take CAM. Satisfaction with alternative medicine and, in females, physical activity, age and level of education are independent predictors for use of CAM. Almost half of these patients do not inform their physician regarding the use of this medication.

The prevalence of use of CAM in CLD in Germany is comparable to another study in the US [12], in which 27% of the patients with CLD reported the use of either herbal medicine, dietary supplements or homeopathy. However, the prevalence is much lower than in other studies concerning CAM use in CLD patients (up to 80%), including mainly non-Western populations [16, 19, 20, 31, 32]. Although the differences in the prevalence maybe due to cultural differences, most of these studies also included mind-body based therapies and prayers among CAM, which were not evaluated in depth in the present study and therefore might be underrepresented.

The most commonly taken form of herbal remedy in our study was milk thistle, which is similar to other studies, where milk thistle was taken by up to 18% of patients [16, 19]. Milk thistle is thought to have hepatoprotective properties and its use is considered to be safe [33]. Clinical trials in patients with chronic hepatitis C (non-responders to interferon) and in patients with NAFLD with milk-thistle have been performed, however, they failed to show any significant beneficial effects of milk-thistle in these settings [34, 35].

CAM use in CLD patients has been linked to higher levels of income and education, etiology of CLD, history of hospitalization, disease severity and poorer rating of general health and vitality, female sex, anxiety and age [12, 16–20]. In the present study, an association between use of CAM and middle age in females was observed, which is similar to a study by Fjaer et al., in which use of CAM was highest in the age group from 45 to 64 years [14]. We could find no association between use of CAM and level of education in our study. Higher levels of education have been linked to CAM use [6, 12, 14, 16–18], a possible explanation for this being educated patients being more likely to read up on possible treatments for their illness, to question the doctor´s authority, and wanting to be in control of their own lives [36].

Pursuit of physical exercise also was an independent factor for CAM use in females in the logistic regression analysis. CAM are often used to improve health and support ongoing therapies [16, 20, 31]. In men, taking no further measures for personal health was significantly associated to a decreased use of CAM in the univariate analysis. In their study, Coughlan et al. found patients with hepatitis C and a smoking habit to be less likely to use CAM than non- smokers [19]. This suggests that CAM use is perceived as part of an approach to proactively promote general health and, conversely, use of CAM decreases when no further measures to improve one´s health are taken.

A high score for agreeableness in the “Big five” score was found to be significantly linked to CAM use in men with CLD in the univariate analysis. The trait of agreeableness has been related to the pursuit of a health- enhancing behavior such as healthy eating and exercise [37, 38] and has been significantly linked to both consulting CAM practitioners [39] and greater satisfaction with health care [40–42].

Use of CAM is not necessarily a symptom of dissatisfaction with conventional medicine [36] and both users or non- users of CAM have positive attitudes toward conventional medicine [16], which was also the case in this study, as patients do not regard CAM as an alternative to conventional medicine but as a form of control and coping over health issues [15, 16]. Using CAM as a form of coping with health issues could also explain why significantly more patients prescribed Ursodeoxycholic acid were found to take CAM. Ursodeoxycholic acid is given to patients with primary biliary cirrhosis or primary sclerosing cholangitis for symptom control, however as to date no curative option for both diseases exists, whereby use of CAM would provide patients with the possibility of pro- actively ameliorating their condition.

More than half of the patients in our study did not inform their family doctor about using CAM, which reflects similar results from other studies, in which non- disclosure rates were as high as 72% [17, 43]. The most common reasons for nondisclosure in our study were failure of the healthcare provider to specifically ask about CAM and the patient´s misconception that the healthcare provider would not find this information important. Reasons for non- disclosure of CAM use to the physicians have been reported to be fear of a negative response from their healthcare provider, the medical practitioner not asking actively about CAM use, the assumption that practitioners of conventional medicine have no knowledge about CAM and the patient´s perception that CAM are irrelevant to the existing biomedical treatment [43–45]. However, CAM can be primarily hepatotoxic or interact with medication taken and substances devoid of hepatotoxicity when taken on their own may be harmful for the liver when combined with other potentially hepatotoxic substances [46]. As many herbal products can be purchased without any prescription, are considered to be natural or are readily available (e.g. supermarkets), users might assume them to be harmless. Furthermore, some products are not subjected to testing of quality whereby quantity and quality of the ingredients can be compromised or even toxic and contaminants contained in the herbal preparation, rather than the herbal preparations themselves, in turn might lead to side effects [46]. Therefore, patients should not only be pro- actively asked about their use of CAM by attending doctors but attending doctors should also inform themselves about CAM and their benefits and potential side effects.

There are several limitations to this study. Firstly, although precautions were taken to rule out any bias in the setting of the patient interviews, the face- to-face interviews might still have influenced patients’ answers, especially concerning the topic of satisfaction with medical treatment. Secondly, although most patients completed the questionnaire, not all responded to the sections “satisfaction with medicine”, HADS and MHLC, however there were no differences in baseline characteristics between groups. Despite these limitations, we believe this study gives a better insight into the associations with and frequency of CAM use in CLD patients in Germany.

In conclusion, CAM use is prevalent among CLD patients in Germany and is associated to satisfaction with alternative medicine and active pursuit of improvement of one´s health. However, disclosure rates of CAM use can be very low and attending doctors should specifically inquire about its use.

The datasets used and analyzed during the current study are available from the corresponding author on reasonable request.

## Electronic supplementary material

Below is the link to the electronic supplementary material.

Supplementary Material 1: Questionnaire

Supplementary Material 2: Baseline characteristics among those who did and did not answer the questionnaire

Supplementary Material 3: Number of missing cases for each variable

Supplementary Material 4: Type of CAM taken

## Data Availability

The datasets used and analyzed during the current study are available from the corresponding author on reasonable request.

## Abbreviations

CAM: Complementary and alternative medicine
CLD: Chronic liver disease
GGT: Gamma- glutamyltransferase
HADS: Hospital Anxiety and Depression Score
MHLC: Multidimensional Health Locus of Control
SD: Standard deviation
IQR: Interquartilic range
MASLD: Metabolic Dysfunction Associated Steatotic Liver Disease
MetALD: MASLD and increased alcohol intake
ALD: Alcohol- associated Liver Disease
OR: Odds ratio
CI: Confidence interval
