# Supplementary figures and images for: Use of complementary and alternative medicine in patients with chronic liver diseases in Germany- a multicentric observational study
Source: BMC Complement Med Ther. 2024 Sep 23;24:340. doi: 10.1186/s12906-024-04607-x (PMC11421120; doi:10.1186/s12906-024-04607-x)

**Supplementary file 1; Questionnaire**


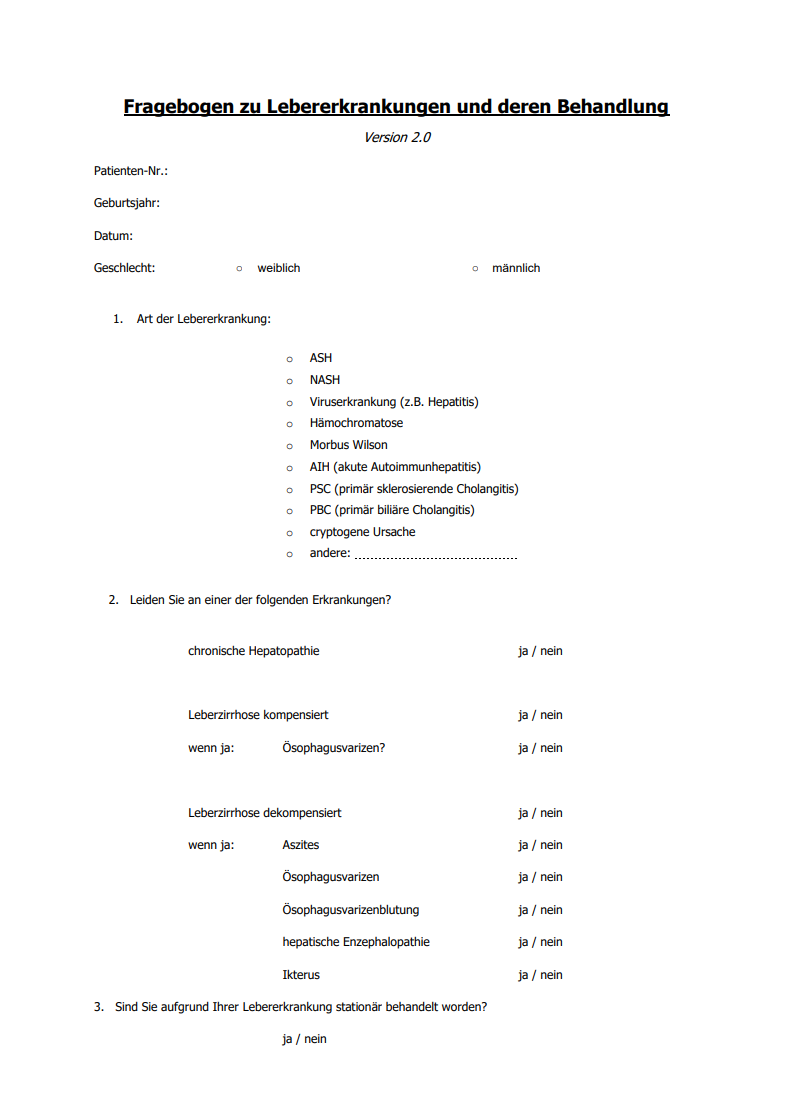

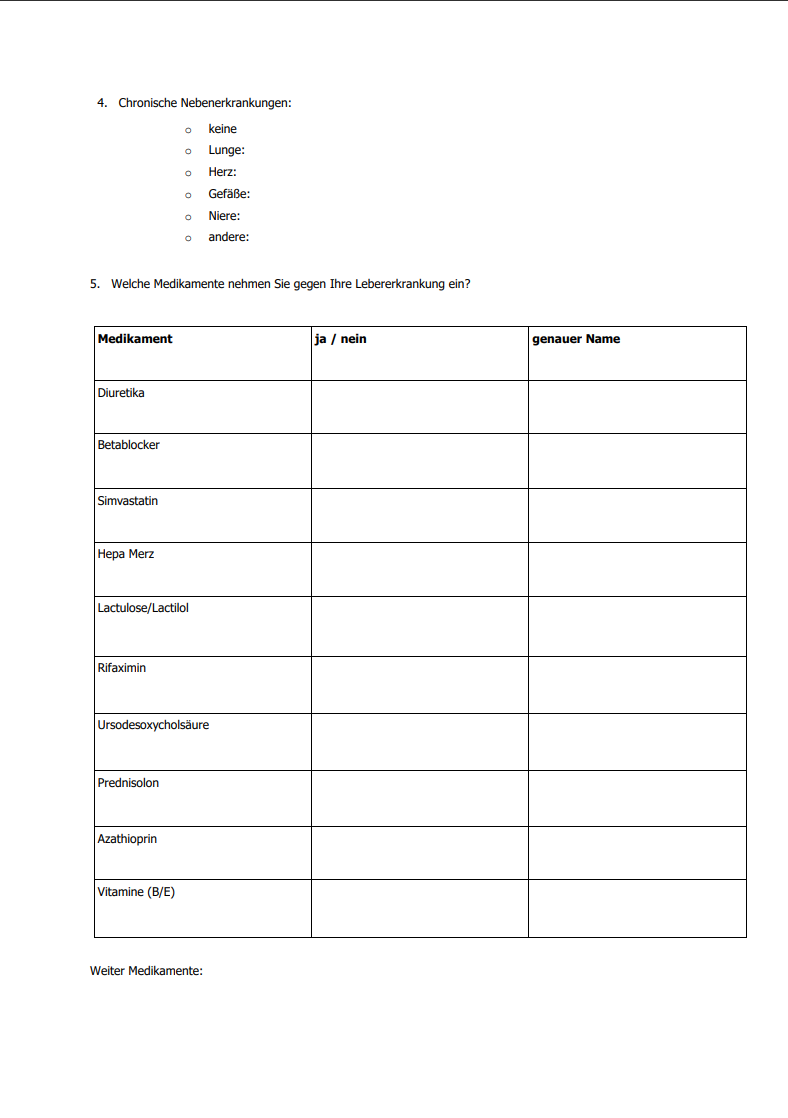


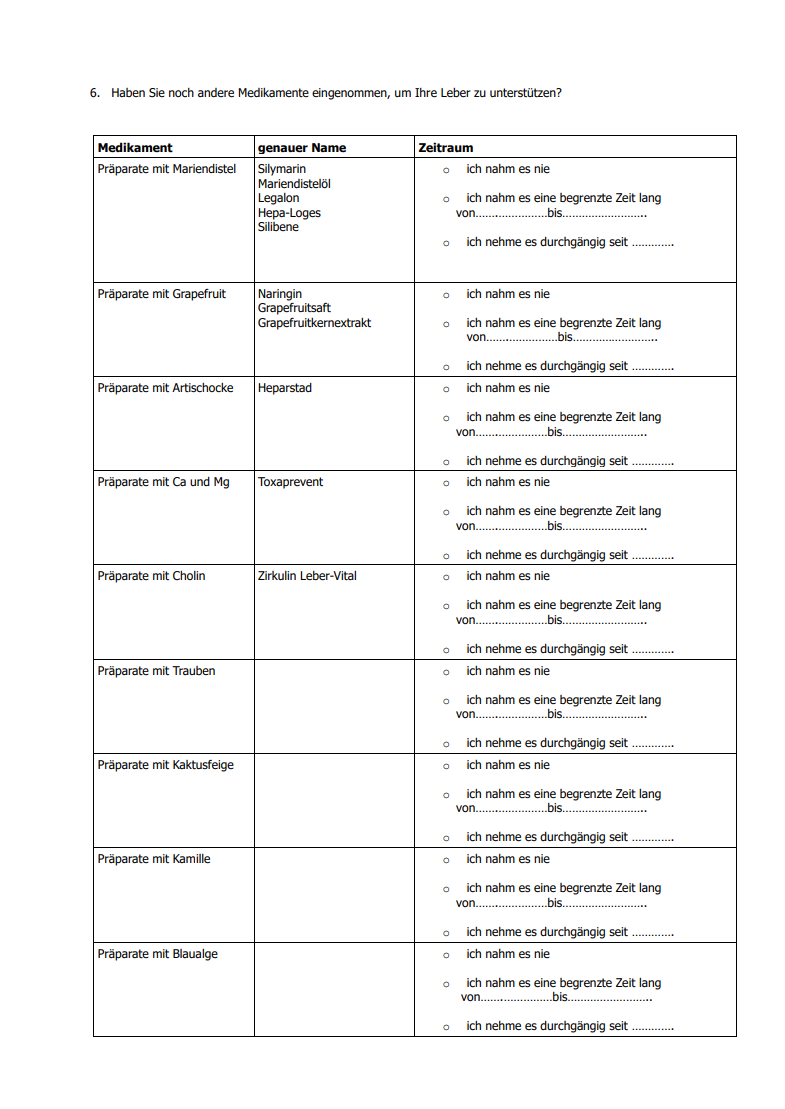

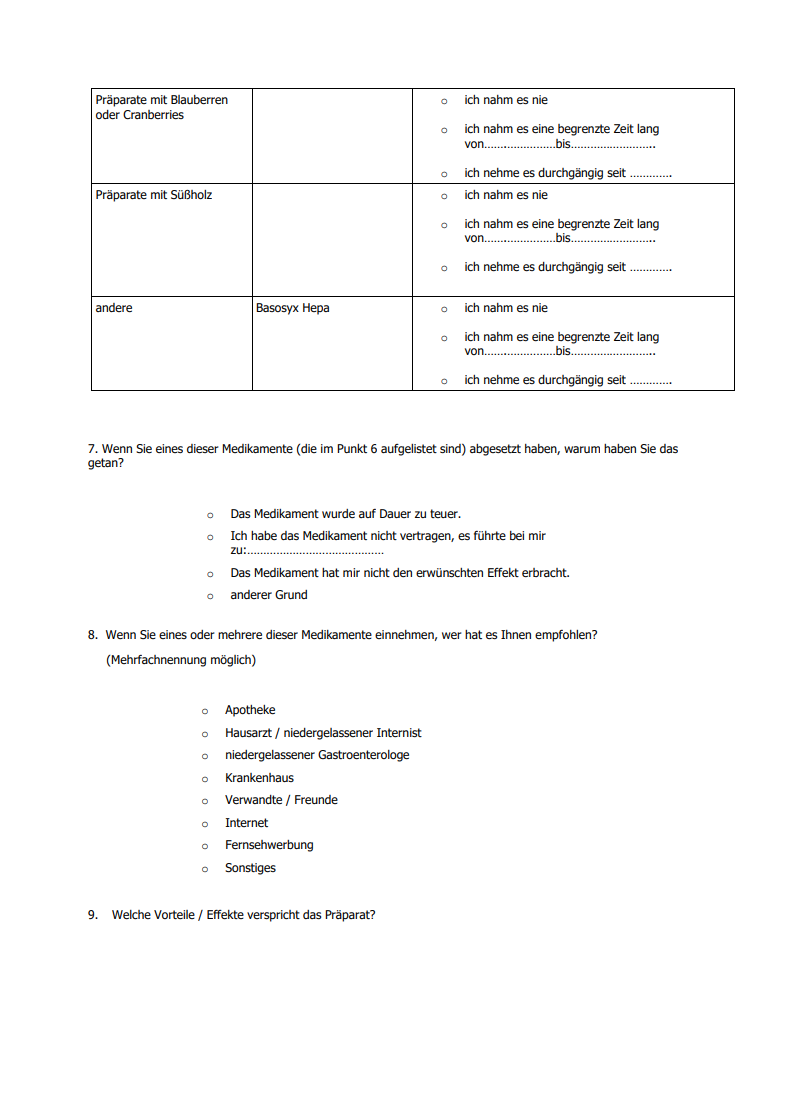


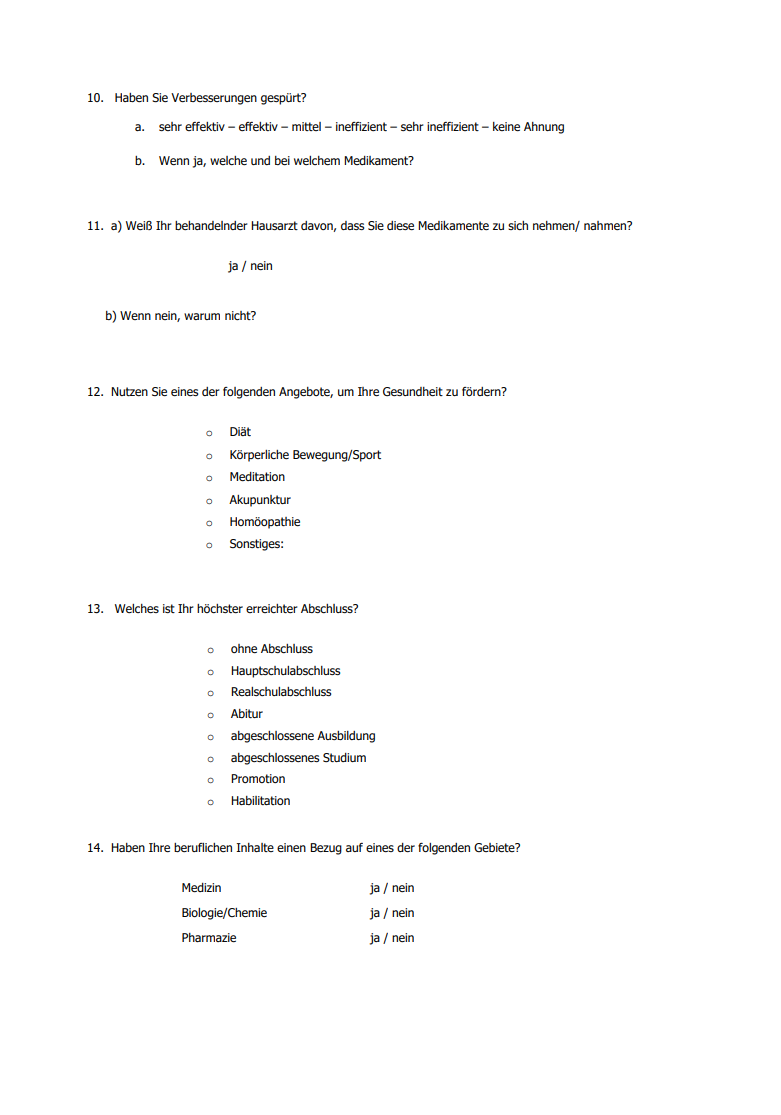

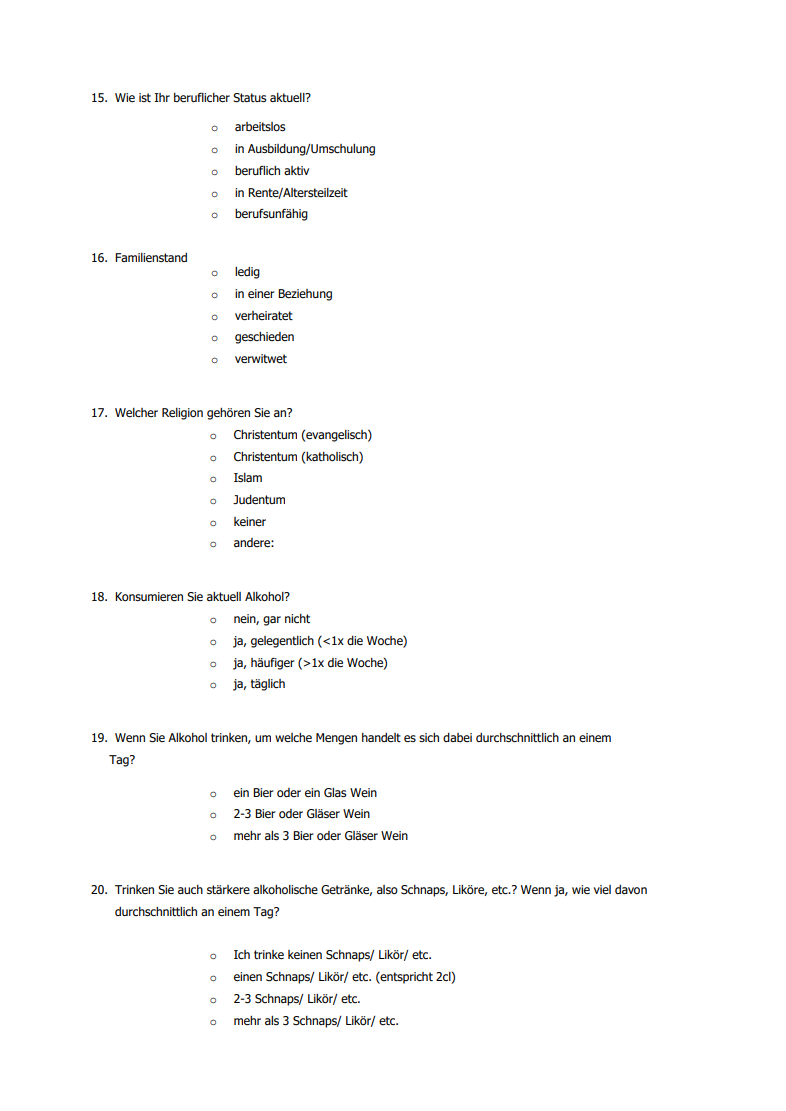


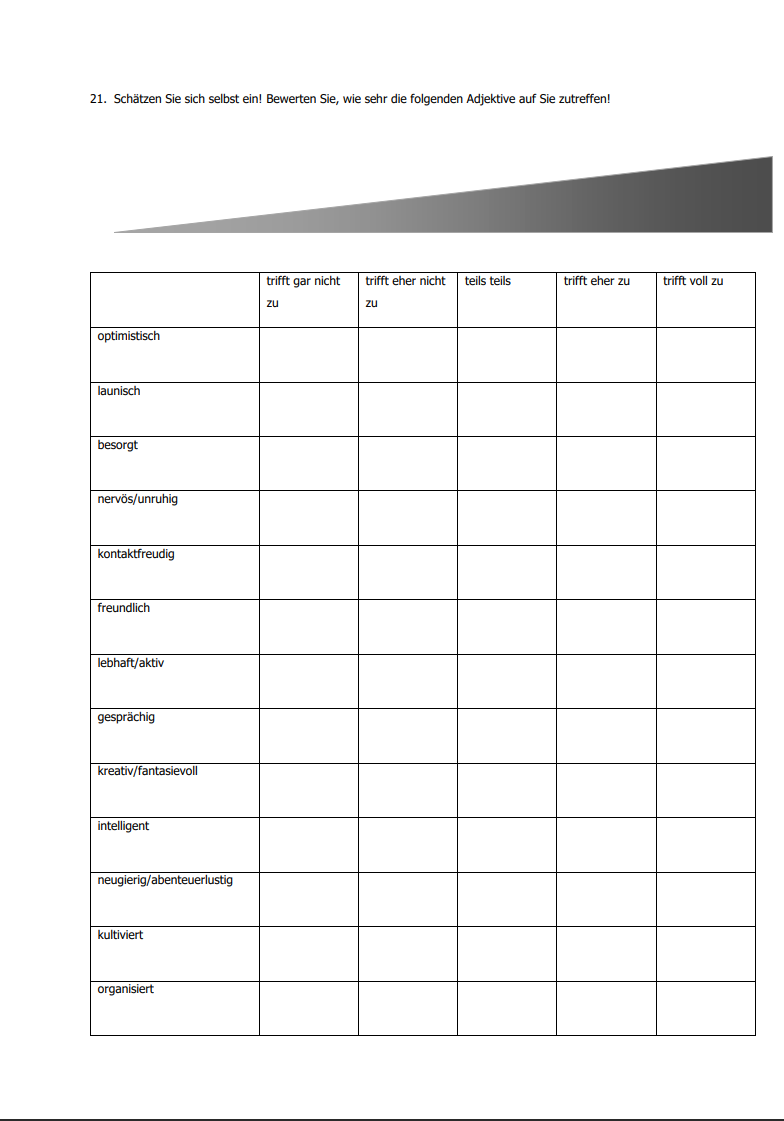

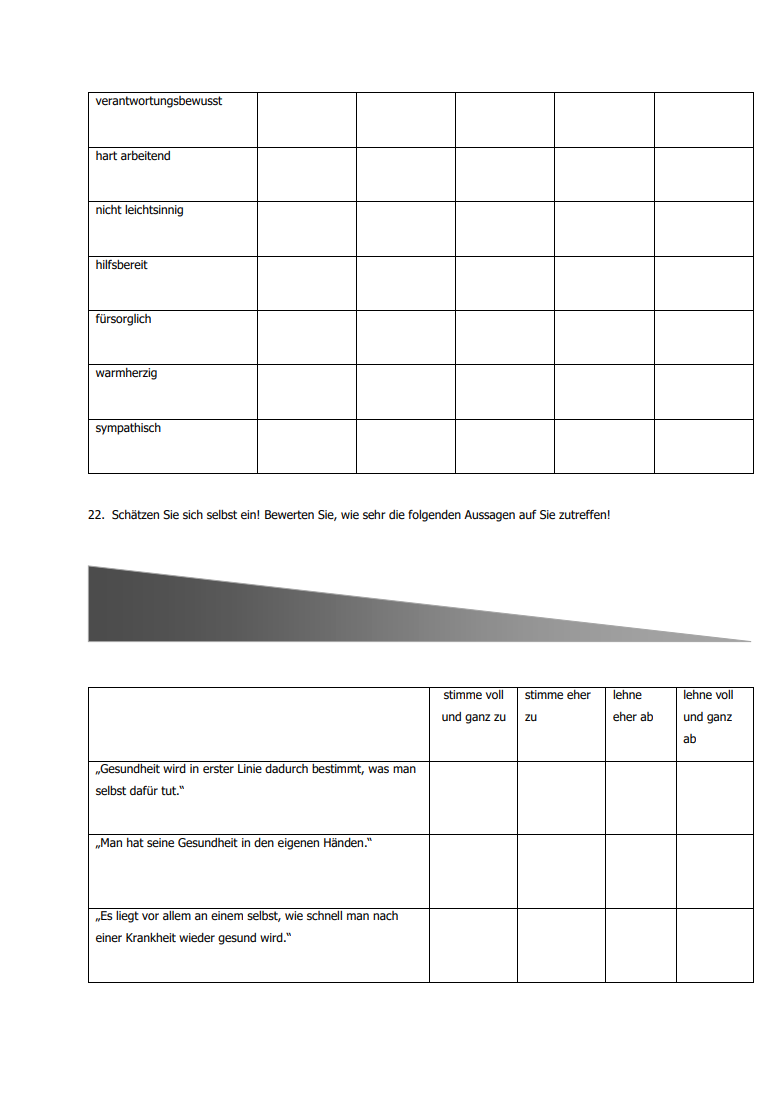


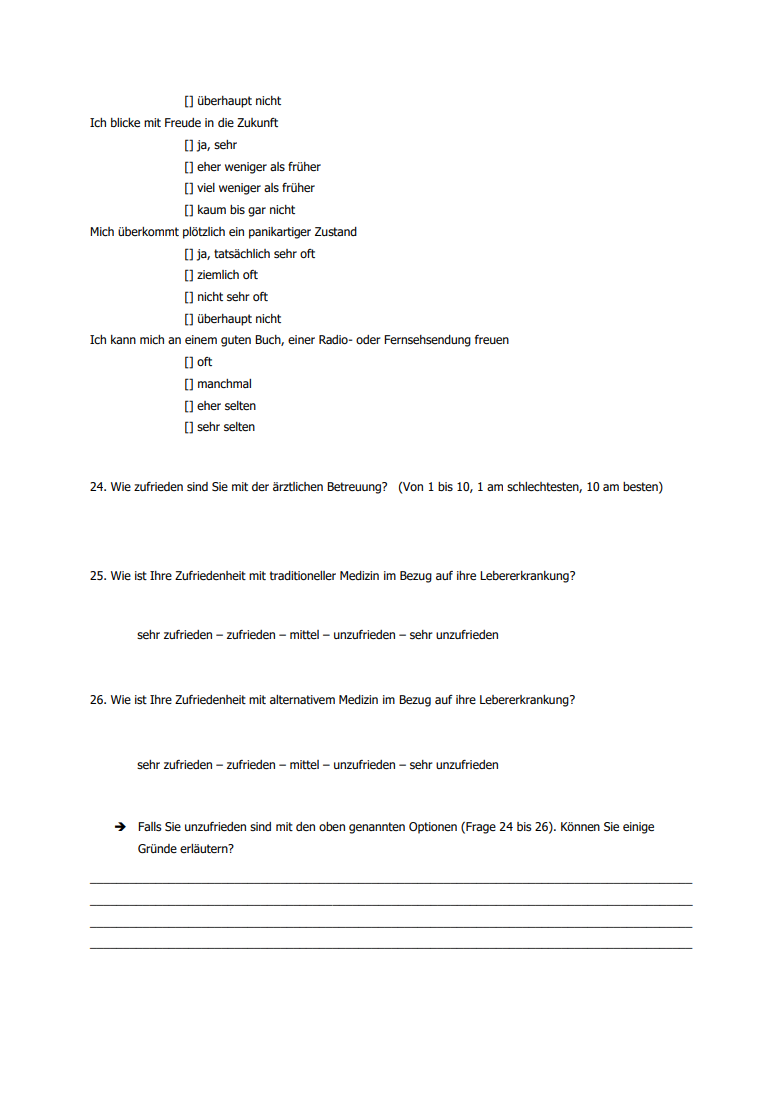

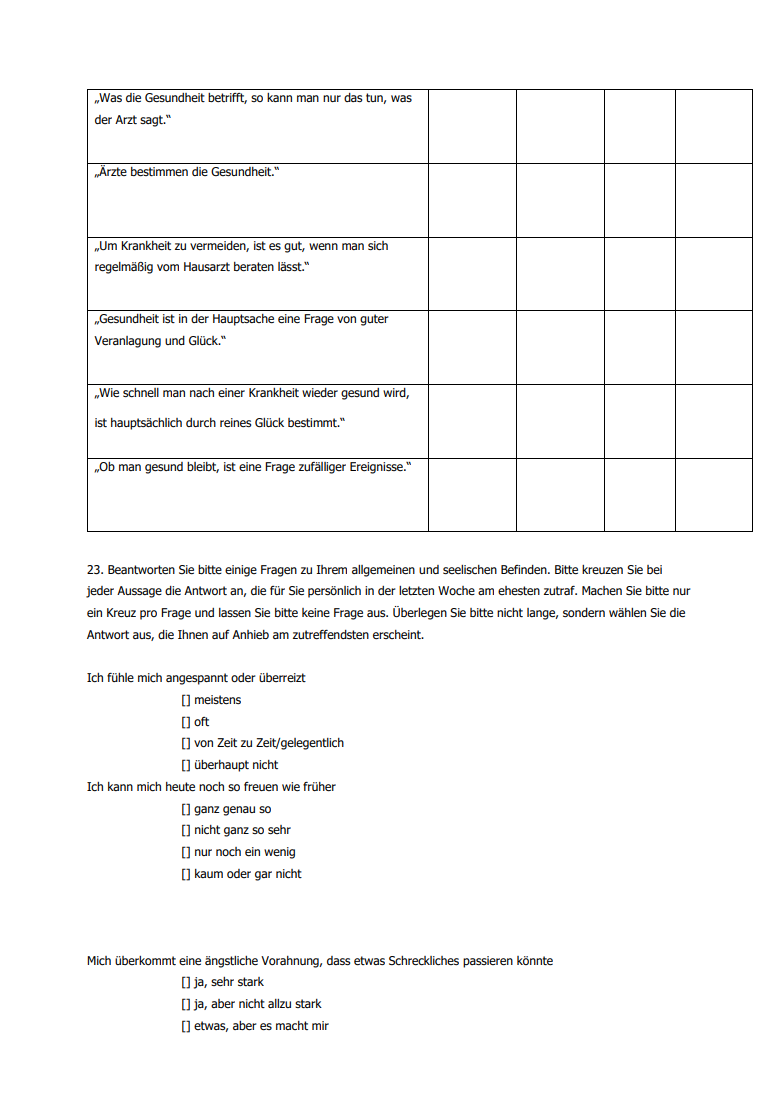

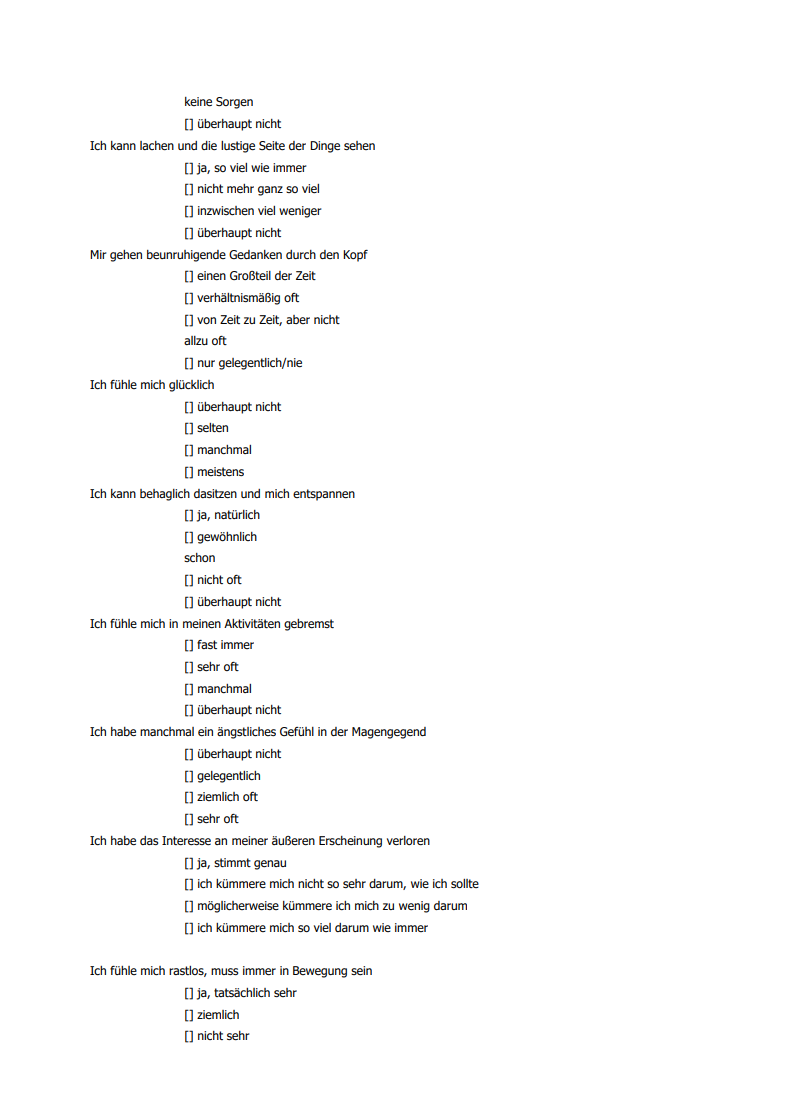

Supplement: Supplementary file 1 — Supplementary Material 1: Questionnaire [file 12906_2024_4607_MOESM1_ESM.docx]
