# Supplementary material for: Use of complementary and alternative medicine in patients with chronic liver diseases in Germany- a multicentric observational study
Source: BMC Complement Med Ther. 2024 Sep 23;24:340. doi: 10.1186/s12906-024-04607-x (PMC11421120; doi:10.1186/s12906-024-04607-x)
Supplement: Supplementary file 2 — Supplementary Material 2: Baseline characteristics among those who did and did not answer the questionnaire [file 12906_2024_4607_MOESM2_ESM.docx]

**Supplementary file 2: Baseline characteristics among those who did and did not answer the questionnaire**

|  | **Answered (n = 227)** | **Answer missing (n = 151)** |
| --- | --- | --- |
| **Age (years)** | Mean 58.0, SD 13.0 | Mean 58.3, SD 13.5 |
| **Halle, n (%)**  **Homburg, n (%)** | 149 (65.6)  78 (34.4) | 151 (100.0)  - |
| **Etiology**  **- AFLD, n (%)**  **- NAFLD, n (%)**  **- Virus, n (%)**  **- Chol., n (%)**  **- Other, n (%)** | 53 (23.3)  29 (12.8)  54 (23.8)  18 (7.9)  73 (32.2) | 44 (29.1)  19 (12.6)  28 (18.5)  8 (5.3)  52 (34.4) |
| **No LC, n (%)**  **Compensated LC, n (%)**  **Decompensated LC, n (%)** | 111 (48.9)  75 (33.0)  41 (18.1) | 70 (46.4)  41 (27.2)  40 (26.5) |
| **OV, n (%)** | 59 (26.0) | 52 (34.4) |
| **Variceal bleeding, n (%)** | 12 (5.3) | 13 (8.6) |
| **Ascites, n (%)** | 39 (17.2) | 37 (24.5) |
| **HE, n (%)** | 8 (3.5) | 10 (6.6) |
| **Jaundice, n (%)** | 9 (4.0) | 15 (9.9) |
| **In- patient, n (%)** | 163 (71.8) | 116 (76.8) |
| **Further comorbidities, n (%)** | 165 (72.7) | 135 (89.4) |
| **Level of education,**  **- No/ basic education, n (%)**  **- High school degree, n (%)**  **- College degree, n (%)** | 4 (1.8)  172 (75.8)  51 (22.5) | 2 (1.3)  120 (79.5)  29 (19.2) |
| **Currently in relationship, n (%)** | 155 (68.3) | 107 (71.3) |
| **Religion**  **- No, n (%)**  **- Christian, n (%)**  **- Other, n (%)** | 134 (59.0)  90 (39.6)  3 (1.3) | 121 (80.1)  28 (18.5)  2 (1.3) |
| **Alcohol consumption present, n (%)** | 64 (28.2) | 39 (25.8) |
| **Further measures for health**  **- Diet, n (%)**  **- PE, n (%)**  **- Mental, n (%)**  **- Homeopathy, n (%)**  **- Nothing, n (%)** | 77 (33.9)  122 (53.7)  15 (6.6)  22 (9.7)  71 (31.4) | 68 (45.0)  80 (53.0)  6 (4.0)  14 (9.3)  27 (17.9) |
| **CAM user** | 48 (21.1) | 44 (29.1) |

**Supplementary file 2:** Comparison of the baseline characteristics among those who did and did not answer the questionnaire. Abbreviations: AFLD: alcoholic fatty liver disease; NAFLD: non- alcoholic fatty liver disease; Virus: liver disease caused by viruses; Chol.: cholestatic liver disease (Primary Biliary Cholangitis, Primary Sclerosing Cholangitis); Other: Autoimmune liver disease, Haemochromatosis, Cryptogenic liver cirrhosis, Cirrhose cardiaque etc.; CLD: chronic liver disease; LC: liver cirrhosis; OV: oesophageal varices; HE: hepatic encephalopathy; Mental: any further measures involving mind- related procedures (e. g. meditation); PE: physical exercise; CAM: complementary and alternative medicine.
