# Supplementary material for: Use of complementary and alternative medicine in patients with chronic liver diseases in Germany- a multicentric observational study
Source: BMC Complement Med Ther. 2024 Sep 23;24:340. doi: 10.1186/s12906-024-04607-x (PMC11421120; doi:10.1186/s12906-024-04607-x)
Supplement: Supplementary file 3 — Supplementary Material 3: Number of missing cases for each variable [file 12906_2024_4607_MOESM3_ESM.docx]

**Supplementary file 3: Number of missing cases for each variable.**

| **Variable** | **Number of valid cases** | **Number of missing cases** |
| --- | --- | --- |
| Further comorbidities | 376 | 2 |
| No further measures for health | 377 | 1 |
| Status of relationship | 377 | 1 |
| Satisfaction with medical treatment in general | 227 | 151 |
| Satisfaction with conventional medicine | 224 | 154 |
| Satisfaction with alternative medicine | 227 | 151 |
| MHLC Score, Internal | 227 | 151 |
| MHLC Score, Powerful others | 227 | 151 |
| MHLC Score, Chance | 227 | 151 |
| HADS Score, Anxiety | 227 | 151 |
| HADS Score, Depression | 227 | 151 |

**Supplementary file 3:** Summary of the number of missing cases for each variable; the total number of cases was n= 378 patients. Only variables with missing cases are listed. Abbreviations: Abbreviations: HADS: Hospital Anxiety and Depression Score; MHLC: Multidimensional Health Locus of Control.
