# Supplementary material for: Use of complementary and alternative medicine in patients with chronic liver diseases in Germany- a multicentric observational study
Source: BMC Complement Med Ther. 2024 Sep 23;24:340. doi: 10.1186/s12906-024-04607-x (PMC11421120; doi:10.1186/s12906-024-04607-x)
Supplement: Supplementary file 4 — Supplementary Material 4: Type of CAM taken [file 12906_2024_4607_MOESM4_ESM.docx]

**Supplementary file 4: Type of CAM taken**

**Supplementary file 4:** CAM use in total, n (%): 92/378 (24.3%). Only substances with recorded use are displayed. No use was recorded for choline, grape seed extract, prickly pear, chamomile and microalgae. Abbreviations: CAM: complementary and alternative medicine; LOA: L- ornithine aspartate; Berries: Bilberries, Goji berries.
